# Using an expiratory resistor, arterial pulse pressure variations predict fluid responsiveness during spontaneous breathing: an experimental porcine study

**DOI:** 10.1186/cc7760

**Published:** 2009-03-20

**Authors:** Michael K Dahl, Simon T Vistisen, Jacob Koefoed-Nielsen, Anders Larsson

**Affiliations:** 1 Anaesthesia and Intensive Care Medicine, North Denmark Region, Aalborg Hospital – Aarhus University Hospitals, Hobrovej 18-22, DK-9000 Aalborg, Denmark

## Abstract

**Introduction:**

Fluid responsiveness prediction is difficult in spontaneously breathing patients. Because the swings in intrathoracic pressure are minor during spontaneous breathing, dynamic parameters like pulse pressure variation (PPV) and systolic pressure variation (SPV) are usually small. We hypothesized that during spontaneous breathing, inspiratory and/or expiratory resistors could induce high arterial pressure variations at hypovolemia and low variations at normovolemia and hypervolemia. Furthermore, we hypothesized that SPV and PPV could predict fluid responsiveness under these conditions.

**Methods:**

Eight prone, anesthetized and spontaneously breathing pigs (20 to 25 kg) were subjected to a sequence of 30% hypovolemia, normovolemia, and 20% and 40% hypervolemia. At each volemic level, the pigs breathed in a randomized order either through an inspiratory and/or an expiratory threshold resistor (7.5 cmH_2_O) or only through the tracheal tube without any resistor. Hemodynamic and respiratory variables were measured during the breathing modes. Fluid responsiveness was defined as a 15% increase in stroke volume (ΔSV) following fluid loading.

**Results:**

Stroke volume was significantly lower at hypovolemia compared with normovolemia, but no differences were found between normovolemia and 20% or 40% hypervolemia. Compared with breathing through no resistor, SPV was magnified by all resistors at hypovolemia whereas there were no changes at normovolemia and hypervolemia. PPV was magnified by the inspiratory resistor and the combined inspiratory and expiratory resistor. Regression analysis of SPV or PPV versus ΔSV showed the highest *R*^2 ^(0.83 for SPV and 0.52 for PPV) when the expiratory resistor was applied. The corresponding sensitivity and specificity for prediction of fluid responsiveness were 100% and 100%, respectively, for SPV and 100% and 81%, respectively, for PPV.

**Conclusions:**

Inspiratory and/or expiratory threshold resistors magnified SPV and PPV in spontaneously breathing pigs during hypovolemia. Using the expiratory resistor SPV and PPV predicted fluid responsiveness with good sensitivity and specificity.

## Introduction

It may be difficult to assess whether a spontaneously breathing patient would hemodynamically benefit from intravenous fluid administration [1,2]. The oldest and most common procedure is observing whether blood pressure will drop by an upright tilt test – and the reverse to this procedure, leg raising, has recently been shown to accurately predict fluid responsiveness [3-5]. This procedure should be performed passively, however, and it is therefore not possible to perform with all beds or stretchers [4,5]. Static measures such as the central venous pressure or the pulmonary artery wedge pressure, if not extremely low, are not useful for assessment of fluid responsiveness [6-8]. A fluid challenge may tip patients with borderline cardiac insufficiency into an overt pulmonary edema, necessitating ventilatory support.

During controlled mechanical ventilation using relatively large tidal volumes with the patient deeply sedated and muscle-relaxed, dynamic measures such as pulse pressure variation (PPV) and systolic pressure variation (SPV) predict fluid responsiveness well [8-10]. These variations are caused by tidal changes in the intrathoracic pressure induced by positive pressure ventilation. During spontaneous breathing the changes in intrathoracic pressures are minimal and often the normal increase in arterial pressure during expiration is difficult to discern [11]. In pathological situations where the left heart filling is hampered during inspiration, such as cardiac tamponade, or when the right heart filling is reduced during expiration by high intrathoracic pressure, for example at acute exacerbation of chronic obstructive lung disease or asthma, however, the normal respiratory variations in arterial pressure may be enhanced, creating pulsus paradoxus [11,12]. In addition, pulsus paradoxus has been reported as a sign of severe hemorrhagic shock [12].

We hypothesized that a low level of expiratory resistance – reducing right heart filling and, some beats later (during the inspiratory phase), reducing the left ventricular stroke volume (SV) – or an inspiratory resistance – enhancing the right heart filling and, some beats after, enhancing the left ventricular SV – could induce high arterial pressure variations at hypovolemia and low arterial pressure variations at normovolemia and hypervolemia. The SPV or PPV might therefore predict fluid responsiveness during spontaneous breathing when expiratory and/or inspiratory resistances are used. In addition, we hypothesized – because an expiratory resistance would theoretically give similar changes as repeated short Valsalva maneuvers (that is, initial augmentation of the arterial pressure followed by a depression) – that tidal changes in arterial pressure caused by an expiratory resistor might give similar or better information about fluid responsiveness than an inspiratory resistor or an inspiratory/expiratory resistor.

The aim of this study was to test in a porcine experimental model whether the SPV and the PPV would be magnified by an expiratory resistor, an inspiratory resistor or a combined inspiratory/expiratory resistor during hypovolemia, normovolemia and hypervolemia, and to test whether the SPV or PPV when using an expiratory resistor would predict the hemodynamic effect of subsequent fluid loading.

## Materials and methods

The study was approved by the national animal ethics committee, and the National Institutes of Health principles of laboratory care were followed. Eight pigs, weighing 25 to 30 kg, were premedicated with apazerone 80 mg intramuscularly and midazolam 10 mg intramuscularly. Anesthesia was induced by remifentanil 1 μg/kg intravenously and propofol 3 mg/kg intravenously. A tracheotomy was performed and the trachea was intubated with a Portex 9.0 ID tube (Smiths Medical, London, UK). The lungs were ventilated by a Servo 900 C ventilator (Siemens-Elema, Solna, Sweden) with volume control, tidal volume of 8 ml/kg, positive end-expiratory pressure of 5 cmH_2_O and a fraction of inspired oxygen of 1.0. The inspiratory time was 35%, the end-inspiratory pause time was 10% and the ventilatory rate was adjusted to achieve an arterial pH of approximately 7.4. Anesthesia was maintained with ketamine 10 mg/kg/hour, remifentanil 0.5 μg/kg/hour and propofol 10 mg/kg/hour. Ringer's acetate 20 ml/kg was infused during the instrumentation phase. In one animal, a bolus of Ringer's acetate 10 ml/kg was administered to stabilize circulation before the main experiment. Monitoring with electrocardiography and pulse oximetry (placed on the tail) was initiated.

Catheters were placed in the right carotid artery, in a femoral artery, and in the right internal jugular vein for sampling of blood gases, monitoring of intravascular pressures and obtaining the pulse contour cardiac output. A pulmonary artery catheter (Swan-Ganz CCO mbo CCO/SvO_2 _7.5 Fr; Edwards Lifescience, Irvine, CA, USA) was placed via the right external jugular vein to monitor the pulmonary artery and central venous pressures. A suprapubic urinary catheter was inserted for monitoring diuresis.

An air-filled 6 Fr catheter was inserted in the tracheal tube with the end-hole 1 cm below the distal opening of the tracheal tube for airway pressure monitoring. The distal esophageal pressure was measured via a latex balloon catheter (Viasys Healthcare, Hochberg, Germany) and an adequate position was ensured as previously described [13]. The tracheal and esophageal catheters were connected to transducers (Edwards Lifesciences) and the signals were transferred to a monitor (S/5 Avance Carestation; GE Healthcare, Chalfont St Giles, UK).

The pulse contour cardiac output was obtained through the catheter (Pulsiocath, 4 Fr, 16 cm; Pulsion Medical Systems, Munich, Germany) placed in a femoral artery connected to the PiCCO monitor (Pulsion Medical Systems). The pulse contour cardiac output measurement was calibrated in triple with the transpulmonary arterial thermodilution technique using cold saline injectate (3 × 10 ml) immediately after induction of anesthesia and before each measurement sequence. In addition, the intrathoracic blood volume and PPV were obtained from the PiCCO device.

During the entire study period, electrocardiography, the cardiac output, blood pressures, the heart rate, and the airway and esophageal pressures were recorded continuously for later analyses. Blood gases were sampled from the right carotid and the pulmonary artery and were analyzed by an ABL 710 (Radiometer, Copenhagen, Denmark).

### Experimental protocol

**Figure 1:**
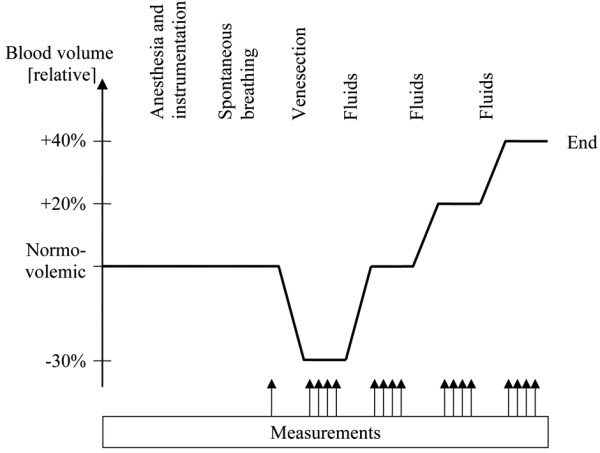
Outline of the experiment. The experimental procedure. Venesection, venesection of 30% of the estimated blood volume. Fluids, intravenous infusion of a starch solution of first 30% and then 20% of the estimated blood volume. Measurements, measurements of hemodynamic and respiratory variables. Tests were performed with the different resistors in a randomized order (see text). End, end of the experiment.

The outline of the experiment is shown in Figure 1. After instrumentation, the animal was placed prone and an interval of 20 minutes was allowed before spontaneous breathing was attempted; the ventilatory rate was reduced to one-half, the triggering level of the ventilator was set at -1 cmH_2_O, the remifentanil infusion was stopped, and the ketamine and propofol infusions were halved and then adjusted to maintain adequate anesthesia (no movement and no reaction to painful stimulation of the anterior toes). When spontaneous breathing attempts began (the animal started to initiate breaths by triggering the ventilator), the ventilator was set to low-level pressure support (2 cmH_2_O above the positive end-expiratory pressure). After about 2 minutes, the animal was connected to a spontaneous breathing device consisting of a Y-piece with inspiratory and expiratory valves and an anesthesia balloon with a valve regulating the oxygen flow from a flowmeter connected to a central pressurized oxygen source. The balloon was attached proximal to the inspiratory valve and the oxygen flow was regulated manually, keeping the balloon slightly expanded but still flaccid. In a bench test, the valves and the Y-piece generated <1 cmH_2_O resistance to inspiratory and expiratory flow.

In the main experiment, when testing the effect of an expiratory resistance, the expiratory valve was replaced with a 7.5 cmH_2_O threshold resistor (CPAP; Philips Respironics, Herrshing, Germany); when the effect of an inspiratory resistance was tested, the inspiratory valve was replaced with a 7.5 cmH_2_O threshold resistor (CPAP; Philips Respironics); and when the effect of both expiratory and inspiratory resistances was tested, both resistors were connected as described above. In a bench test before the experiment, with the connectors used, the inspiratory resistor gave a resistance of 8.5 cmH_2_O and the expiratory resistor gave a resistance of 7.5 cmH_2_O.

Baseline data were obtained during spontaneous breathing without a resistor. Thereafter, the main experiment was initiated. Measurements were performed when the animals breathed without a resistor, with the expiratory resistor, with the inspiratory resistor and with the inspiratory/expiratory resistor at four volemic levels: 30% hypovolemia, normovolemia, and 20% and 40% hypervolemia. The order of breathing modes was randomized by computer randomization. Hypovolemia was achieved by venesection of 30% of the estimated blood volume, normovolemia was achieved by replacing the depleted blood with a starch solution (Voluven; Fresenius Kabi, Uppsala, Sweden), and 20% and 40% hypervolemia were achieved by infusion of corresponding volumes of the starch solution.

The blood volume was estimated as 179 × body weight^(0.73)^, which is about 8% of the body weight [14]. The sequence of intravascular volume levels was always hypovolemia, normovolemia, and 20% and 40% hypervolemia. Each infusion or blood removal was performed over 5 to 10 minutes. During these procedures the animal breathed with pressure support using the settings as described above. This was followed by a 5-minute stabilization period with spontaneous breathing before a new measurement sequence was performed. Electrocardiography, the cardiac output, systolic arterial blood pressures, the heart rate, the pulmonary artery wedge pressure, the central venous pressure, the intrathoracic blood volume, and the SPV and PPV were registered 3 minutes after the resistor change. Blood gases were sampled, and the airway and esophageal pressures were obtained for calculation of the transpulmonary pressure and respiratory intrathoracic pressure variations.

After the experiment, the animal was killed by an overdose of thiopental and potassium chloride intravenously.

### Calculations

Fluid responsiveness was defined as an increase in the SV of 15% after fluid loading.

Before the study, we decided to manually calculate the PPV and the SPV from the pressure tracings, because we have previously found a significant variation in the PiCCO monitor's stated SPV and PPV values during controlled ventilation of pigs [15]. We had problems with measuring the PPV correctly, however, and therefore the PPV was obtained automatically from the PiCCO device. The SPV was calculated over six respiratory cycles as previously described by Michard and colleagues [16].

The SV was obtained as the ratio of cardiac output/heart rate.

Airway pressure variations were calculated as the mean values for six respiratory cycles of maximal airway pressure (expiration) minus minimum airway pressure (inspiration). The same calculations were carried out regarding the pleural (esophageal) pressure. The transpulmonary pressure was obtained as the airway pressure minus esophageal pressure at similar time points, and the variations were registered simultaneously with the airway pressure.

### Statistical analysis

The statistical analyses were performed using the SigmaStat 3.5 program (Systat Inc., Point Richmond, CA, USA). Results are presented as the mean and standard deviation, if not otherwise indicated. *P *< 0.05 was considered significant. Normal distribution of the data was checked with the Kolmogorov–Smirnov test.

The overall changes in cardiac output, SV, central venous pressure and intrathoracic blood volume between the different volemic levels for no resistor were analyzed by one-way analysis of variance and the Tukey test. The overall changes in PPV and SPV between the different volemic levels with the different resistors in place were analyzed by two-way analysis of variance and the Tukey test. The differences in hemodynamics and in respiratory pressures caused by the different resistors at 30% hypovolemia were analyzed by one-way analysis of variance and the Tukey test. The relation between the SV and the SPV or PPV was analyzed by linear regression, and the sensitivity and specificity were calculated by standard formulas after inspection of the receiver operating characteristic curves (SigmaPlot 11.0; Systat Inc.).

## Results

### Hemodynamics without a resistor

The cardiac output, the SV, the central venous pressure and the intrathoracic blood volume were significantly lower during hypovolemia than during normovolemia, whereas there were minor or insignificant changes between the other volemic steps (Table 1). The SPV was similar at all volemic levels, whereas the PPV was significantly higher at -30% hypovolemia (Table 1).

**Table 1:** Central hemodynamics and arterial pressure variations at the four volemic levels

	-30% hypovolemia	0% normovolemia	+20% hypervolemia	+40% hypervolemia
No resistor				
Cardiac output (l/min)	3.2 ± 0.7	7.5 ± 1.6*	7.9 ± 2.0	7.7 ± 2.2
Stroke volume (ml)	24 ± 5	65 ± 11*	63 ± 10	62 ± 10
Central venous pressure (mmHg)	0 ± 2	6 ± 2*	7 ± 2*	8 ± 2*
Intrathoracic blood volume (ml)	485 ± 88	814 ± 177*	849 ± 156	924 ± 213
Central venous oxygen saturation	0.89 ± 0.05	0.99 ± 0.04*	1 ± 0.02	0.98 ± 0.04
Lactate (mmol/l)	1.2 ± 1.3	2.4 ± 1.8	1.9 ± 1.2	1.2 ± 0.8
Base excess (mmol/l)	4.1 ± 1.5	2.2 ± 1.7	2.2 ± 1.6	3.0 ± 1.9
Systolic pressure variation				
No resistor (%)	5 ± 2	3 ± 2	2 ± 1	2 ± 1
Inspiratory resistor (%)	10 ± 5^†^	4 ± 2*	5 ± 2	4 ± 2
Expiratory resistor (%)	11 ± 2^†^	4 ± 2*	4 ± 1	3 ± 2
Inspiratory/expiratory resistor (%)	13 ± 5^†^	5 ± 3*	5 ± 2	4 ± 2
Pulse pressure variation				
No resistor (%)	17 ± 5	12 ± 2*	12 ± 4	12 ± 1
Inspiratory resistor (%)	25 ± 6^†^	16 ± 4* ^†^	16 ± 6 ^†^	15 ± 5^†^
Expiratory resistor (%)	25 ± 6	13 ± 6*	12 ± 3	11 ± 3
Inspiratory/expiratory resistor (%)	26 ± 7^†^	14 ± 6* ^†^	14 ± 5^†^	13 ± 6^†^

Data presented as the mean ± standard deviation. *P < 0.05 compared with the previous volemic level. ^†^*P *< 0.05 compared with no resistor at the same volemic level.

### Effects of resistors on airway and esophageal pressures

The airway and esophageal pressure swings were generally higher with resistors than without a resistor (Table 2). The transpulmonary pressure swings were somewhat higher with the inspiratory/expiratory resistor compared with no resistor, indicating larger tidal volumes.

**Table 2:** Respiratory pressures and hemodynamics at 30% hypovolemia

	No resistor	Inspiratory resistor	Expiratory resistor	Inspiratory/expiratory resistor
Airway pressure (AP)				
Inspiratory (cmH_2_O)	-1 ± 4	-7 ± 2*	-3 ± 4	-5 ± 2*
Expiratory (cmH_2_O)	3 ± 5	1 ± 2	5 ± 2	5 ± 2
ΔAP (cmH_2_O)	4 ± 1	8 ± 1*	8 ± 2*	11 ± 4*
Esophageal pressure (EP)				
Inspiratory (cmH_2_O)	-4 ± 2	-9 ± 3*	-6 ± 3	-8 ± 2*
Expiratory (cmH_2_O)	-2 ± 1	-3 ± 3	-1 ± 2	-2 ± 3
ΔEP (cmH_2_O)	3 ± 1	6 ± 1*	5 ± 2*	6 ± 2*
Transpulmonary pressure (TP)				
Inspiratory (cmH_2_O)	3 ± 4	2 ± 4	4 ± 2	3 ± 4
Expiratory (cmH_2_O)	5 ± 5	5 ± 4	6 ± 1	7 ± 3
ΔTP (cmH_2_O)	1 ± 2	3 ± 1	2 ± 1	4 ± 3*
Heart rate (/min)	130 ± 21	133 ± 12	138 ± 18	137 ± 23
Cardiac output (l/min)	3.2 ± 0.7	3.3 ± 0.4	3.3 ± 0.5	3.2 ± 0.5
Stroke volume (ml)	25 ± 5	25 ± 4	24 ± 4	24 ± 5
PAWP during inspiration (mmHg)	-2 ± 5	-7 ± 4	-3 ± 4	-5 ± 3
PAWP during expiration (mmHg)	4 ± 3	6 ± 2	8 ± 2*	7 ± 2
Mean arterial pressure (mmHg)	55 ± 6	59 ± 5	60 ± 7	59 ± 5
Central venous pressure (mmHg)	0 ± 2	-1 ± 3	1 ± 3	1 ± 3

Data presented as the mean ± standard deviation. *P < 0.05 compared with no resistor. PAWP, pulmonary artery wedge pressure.

### Hemodynamic consequences at each volemic level of applying the resistors

At each volemic level, the cardiac output, the SV, the mean arterial pressure and the heart rate did not change when applying the resistors, whereas the swings in pulmonary artery wedge pressure were slightly related to the swings in airway pressure (*R*^2 ^= 0.12) (Table 2).

At 30% hypovolemia, as compared with no resistor, the SPV was magnified by all resistors, whereas no changes were found at normovolemia and at 20% and 40% hypervolemia. The PPV was magnified by the inspiratory resistor and the inspiratory/expiratory resistor (Table 1).

### Correlations between changes in stroke volume and systolic or pulse pressure variation using the different resistors

The regression analyses between the change in SV and the SPV or PPV using the different resistors are presented in Table 3. The *R*^2 ^value was generally higher when the expiratory resistor was applied with the highest correlation (*R*^2 ^= 0.83) for the SPV.

**Table 3:** Correlation of systolic pressure variation and pulse pressure variation versus the change in stroke volume

	Systolic pressure variation	Pulse pressure variation
No resistor	0.37	0.37
Inspiratory resistor	0.45	0.36
Expiratory resistor	0.83	0.52
Inspiratory/expiratory resistor	0.50	0.31

Data presented as *R*^2 ^values obtained by linear regression for systolic pressure variation or pulse pressure variation versus the increase in stroke volume by a subsequent fluid loading with no resistor and with the inspiratory, expiratory, and inspiratory/expiratory resistors.

### Performances of systolic pressure and pulse pressure variations for each resistor

**Figure 2:**
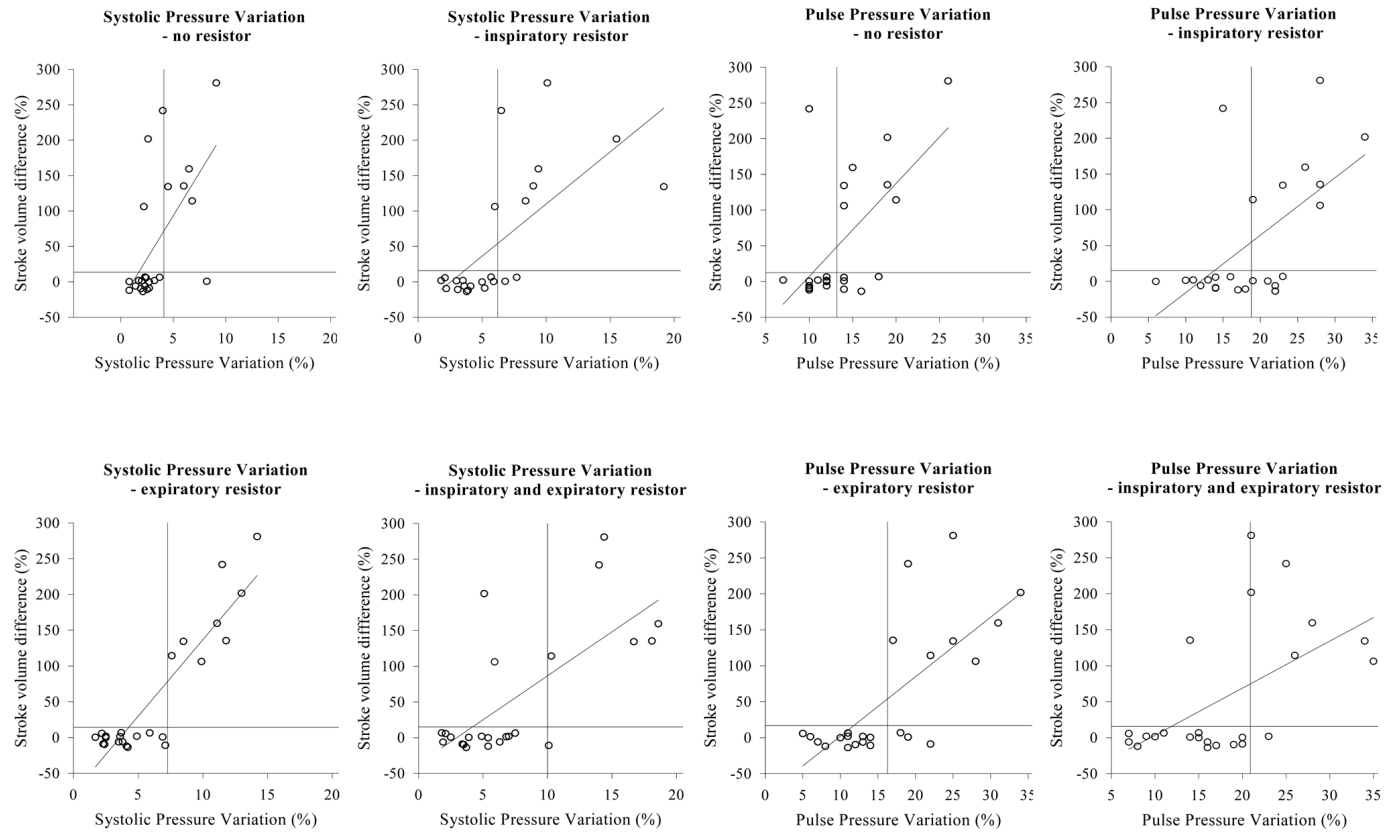
Linear regression for systolic pressure variation and pulse pressure variation. Systolic pressure variation and pulse pressure variation before fluid administration versus the change in stroke volume following fluid loading without and with the expiratory resistor. Regression lines are indicated. All measurement points are used in the regression analyses. Horizontal lines, relevant change in stroke volume (15%); vertical lines, cutoff values used.

**Figure 3:**
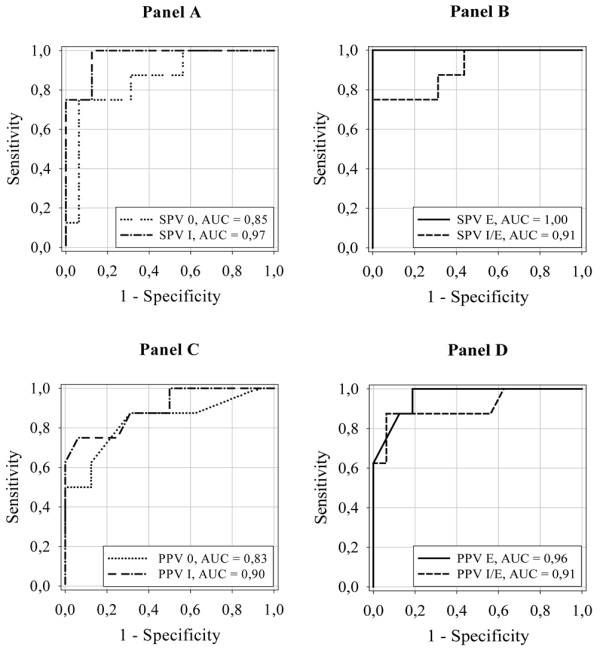
Receiver operating characteristic curves for systolic pressure variation and pulse pressure variation. Receiver operating characteristic curves for **(a), (b) **systolic pressure variation and **(c), (d) **pulse pressure variation, with the four different respiratory interventions, for predicting a 15% increase in stroke volume by subsequent fluid loading. SPV 0, systolic pressure variation with no resistor; SPV I, systolic pressure variation with the inspiratory resistor; SPV E, systolic pressure variation with the expiratory resistor; SPV I/E, systolic pressure variation with the combined inspiratory and expiratory resistor; PPV 0, pulse pressure variation with no resistor; PPV I, pulse pressure variation with the inspiratory resistor; PPV E, pulse pressure variation with the expiratory resistor; PPV I/E, pulse pressure variation with the combined inspiratory and expiratory resistor; AUC, area under the curve.

Using a 15% increase in SV as the definition of fluid responsiveness, the sensitivity and specificity for SPV and PPV were as shown in Table 4. The highest sensitivity was found for the expiratory resistor. The SPV gave sensitivity and specificity of 100% for a SPV cutoff value of 7% with the expiratory resistor, and sensitivity and specificity of 63% and 94%, respectively, for a cutoff value of 4% without a resistor (Figures 2 and 3). Corresponding values for the PPV were sensitivity and specificity of 100% and 81%, respectively, and sensitivity and specificity of 88% and 69%, respectively, for PPV cutoff values of 16% and 13%, respectively (Figures 2 and 3).

**Table 4:** Sensitivity, specificity, positive and negative predictive values for the pressure variations with different respiratory interventions

	Sensitivity (%)	Specificity (%)	Positive predictive value (%)	Negative predictive value (%)
Systolic pressure variation				
No resistor	63	94	83	83
Inspiratory resistor	88	88	78	93
Expiratory resistor	100	100	100	100
Inspiratory/expiratory resistor	75	94	86	88
Pulse pressure variation				
No resistor	88	69	58	92
Inspiratory resistor	88	69	58	92
Expiratory resistor	100	81	73	100
Inspiratory/expiratory resistor	88	94	88	94

### Central venous oxygen saturation, lactate and blood gases

The central venous oxygen saturation increased from normovolemia, whereas the partial arterial tension of oxygen and the partial arterial tension of carbon dioxide (data not shown) as well as the base excess and lactate were stable during the experiment, with no significant changes between the volemic levels or respiratory modes.

## Discussion

We have shown in this exploratory study in spontaneously breathing pigs that inspiratory and/or expiratory threshold resistors magnified arterial pressure variations markedly during hypovolemia, whereas changes in arterial pressure variations were minor during normovolemia and hypervolemia; that the expiratory resistor gave a better relation between the SPV or PPV and the change in SV by subsequent fluid loading than the inspiratory resistor or the inspiratory/expiratory resistor; and that the SPV and PPV using the expiratory resistor predicted fluid responsiveness with good sensitivity and specificity.

We manipulated the intrathoracic pressure to magnify the normal swings in arterial pressure. This concept has long been used clinically during controlled mechanical ventilation [8-10]. The ventilator-induced cyclic changes in intrathoracic pressure produce significant arterial pressure variations if the circulation is fluid responsive. The tidal volume, however, has to be above 8 ml/kg predicted body weight [17], which is higher than recommended in critically ill, ventilated patients [18]. Furthermore, the patient should have normal right heart function, no atrial fibrillation, and no spontaneous breathing activity [8-10]. Indeed, if the patient is breathing in a spontaneous ventilator mode, the arterial pressure variations will not give any information about fluid responsiveness [19].

In spontaneously breathing, hemodynamically unstable patients, Soubrier and colleagues found a sensitivity and specificity for predicting the effect of a subsequent fluid administration of 63% and 92%, respectively, for the PPV, and a sensitivity and specificity of 47% and 92%, respectively, for the SPV – as discussed in the accompanying editorial [20] – agreeing well with our results without resistors. Our study therefore confirms that arterial pressure variations during normal spontaneous breathing are not useful for fluid responsiveness prediction, mainly because of low sensitivity. Soubrier and colleagues also investigated whether a forceful inspiration and expiration (with no resistance) would improve the ability of the SPV and the PPV to predict fluid responsiveness [21]. The sensitivity was even lower, however, with this maneuver [21]. Indeed, we found a somewhat lower sensitivity with the expiratory/inspiratory resistor for SPV than with the other resistors.

In the editorial to the paper by Soubrier and colleagues, de Backer and Pinsky discussed whether manipulation of the intrathoracic pressure by a Valsalva maneuver – that is, a forceful expiration against a resistance – could be used to generate arterial pressure variations that could predict fluid responsiveness [20]. In fact, this has now been shown in a very recent study by Garcia and colleagues [22]. A Valsalva maneuver causes an immediate increase in cardiac output by squeezing blood from the pulmonary circulation to the left heart, but this is very quickly followed by a marked reduction in cardiac output due to reduced right heart filling [23]. As the Valsalva maneuver may induce a pronounced drop in blood pressure during hypovolemia, it may be difficult to perform in a patient distressed by circulatory compromise or pain – and the maneuver may induce changes in the heart rate. Moreover, the Valsalva maneuver may generate quite different intrathoracic pressures dependent on the patient's effort.

On the other hand, breathing against an expiratory resistance could be considered to give short, intermittent Valsalva maneuvers. This will cyclically reduce right heart filling and induce variations in arterial blood pressure that theoretically would be more pronounced when the circulation is fluid responsive. Indeed, in our study when using the expiratory resistor, the SPV was markedly enhanced during hypovolemia and became normalized during normovolemia; in addition, the SPV and the PPV could be used to predict fluid responsiveness. The minor difference in performance between the PPV and the SPV in our study is probably due to differences in obtaining these variables. The PPV was obtained from the PiCCO device and the SPV was obtained manually from the pressure tracings (see Calculations).

The inspiratory resistor and the inspiratory/expiratory resistor did also magnify the arterial pressure variations. Both of these resistors, however, gave inferior precision for fluid responsiveness prediction compared with the expiratory resistor. An explanation could be the different changes in intrathoracic pressures induced by the resistors; the expiratory resistor mainly increases the intrathoracic pressure during expiration, whereas the inspiratory resistor decreases the intrathoracic pressure during inspiration (Table 2). This decrease in the inspiratory intrathoracic pressure decreases left heart filling by reducing the pressure difference between the pulmonary vessels and the left atrium (as reflected in the markedly negative inspiratory pulmonary artery wedge pressure; Table 2), but simultaneously it improves right heart filling and thus, some beats afterwards, improves the left heart filling and the SV. Because of anatomical reasons the caval veins should be more affected by the pleural pressure than by the airway or the transpulmonary pressures, and thus the right heart filling should be dependent on the difference between the vein pressure and the pleural (esophageal) pressure. In fact, the inspiratory resistor reduced the inspiratory esophageal pressure and theoretically improved the right heart filling, whereas the expiratory device increased the expiratory esophageal pressure and theoretically reduced the right heart filling. The inspiratory/expiratory device had a combined effect.

The difference between the inspiratory and inspiratory/expiratory resistors could therefore be explained by the Frank–Starling heart function curve. With an expiratory resistor the filling becomes lower, causing the heart function to work on the steeper left part of the curve; whereas an inspiratory resistor improves filling, causing the heart function to work on the right less steep part of the curve. This would make the pressure variations with the expiratory resistor somewhat higher than with the inspiratory resistor, and the signal would be more pronounced. According to this reasoning, the inspiratory/expiratory resistor – making the heart work on a wider part of the Frank–Starling curve – would give highest pressure variations, agreeing with our result.

Inspiratory resistors have been found to improve cardiac output in experimental settings of hypovolemia [24,25]. We could not confirm this finding. The resistance level used in our study, however, was less than in the studies investigating the effect on cardiac output by inspiratory threshold resistors [24,25].

The use of an expiratory resistor connected to a nose–mouth mask is feasible in the clinic. It is used commonly for breathing physiotherapy in patients in the intensive care unit and in patients before and after surgery [26].

Our study has several limitations and caution should therefore be taken when translating the results to patients. First, we studied a limited number of young healthy animals with normal heart function and with no arrhythmias. Second, because we did not *a priori *know the effect on the arterial pressure variations by hypovolemia and volume challenges, both the hypovolemic level and the volume challenges were substantial and, furthermore, two different volume challenges were used. Third, the level of expiratory resistance used might not be optimal in patients. We chose these resistors because 5 to 10 cmH_2_O is commonly used as expiratory impedance clinically (for example, for positive end-expiratory pressure or continuous positive airway pressure) and are accepted by most patients. Fourth, some values used in the receiver operating characteristic and linear regression analyses were dependent, making these analyses less strong.

## Conclusions

The present exploratory animal study shows that arterial pressure variations predict fluid responsiveness during spontaneous breathing with an expiratory resistor.

## Key messages

• Using an expiratory resistor, fluid responsiveness can be predicted by assessment of arterial pressure variations during spontaneous breathing.

## Abbreviations

PPV: pulse pressure variation; SPV: systolic pressure variation; SV: stroke volume.

## Competing interests

The authors declare that they have no competing interests.

## Authors' contributions

MKD and AL participated in the design, laboratory work, data analyses and writing of the manuscript. STV, JK-N participated in the design, the laboratory work and in the finalizing of the manuscript.
